# Exploring the Role of Community Pharmacists in Obesity and Weight Management in Qatar: A Mixed-Methods Study

**DOI:** 10.2147/RMHP.S309142

**Published:** 2021-06-29

**Authors:** Sawsan AlMukdad, Nancy Zaghloul, Ahmed Awaisu, Ziyad R Mahfoud, Nadir Kheir, Maguy Saffouh El Hajj

**Affiliations:** 1 Weill Cornell Medicine-Qatar, Cornell University, Qatar Foundation - Education City, Doha, Qatar; 2 College of Pharmacy, QU Health, Qatar University, Doha, Qatar; 3 Heart Hospital, Hamad Medical Corporation, Doha, Qatar; 4 College of Pharmacy, Ajman University, Ajman, United Arab Emirates

**Keywords:** weight management, obesity, community pharmacy, pharmacist, Qatar

## Abstract

**Introduction:**

Obesity is a major public health burden in Qatar. Pharmacists can play an important role in providing weight management services (WMSs). This study aimed to explore their attitudes, practice, perceived competence, and role in WMSs in Qatar.

**Methods:**

A mixed-method explanatory sequential design was applied in the study. A validated online questionnaire was administered followed by qualitative individual and focus group interviews.

**Results:**

Two-hundred seventy community pharmacists completed the survey (response rate 45%). More than half of them indicated that they often or always explain to patients the risks associated with overweight and obesity (56.2%), recommend weight loss medications, herbs or dietary supplements (52.4%), and counsel about their proper use and/or side effects (56.9%). Conversely, the majority of the pharmacists rarely or never measure patients’ waist circumference (83.8%) or calculate their body mass index (72.1%). Over 80% had very positive attitudes towards their role in weight management. Around three-quarters of the participants agreed or strongly agreed that difficulty in following-up with patients (80.7%), lack of private consultation area (75.7%), and lack of pharmacist’s time (75.2%) are barriers for implementing WMSs. More than 60% stated that they are fully competent in 7 out of 24 WMSs listed. Some themes generated include pharmacist’s role and impact in weight management, need for training about weight management, and impact of social media on patients’ perceptions.

**Conclusion:**

Qatar community pharmacists reported positive attitudes towards the provision of WMSs. However, they identified several barriers against provision of WMSs. Several strategies are proposed to overcome barriers and to improve the provision of WMSs in community pharmacies in Qatar.

## Introduction

Obesity is a major public health problem and constitutes a significant economic burden worldwide. In 2016, the World Health Organization (WHO) reported a global estimate of more than 1.9 billion overweight adults; of whom, 650 million were obese.^1^ In Middle East and North Africa (MENA) region, the prevalence of overweight and obesity among both adults and children is also alarming with about 66% of females older than 20 years being overweight.^2^ Obesity is associated with a significant risk for developing cardiovascular diseases (CVDs), type 2 diabetes mellitus and cancer.^1^

Healthcare providers can play an important role in educating overweight and obese patients about the optimal strategies for weight reduction and about the risks and benefits of weight loss medications.^3^,^4^ Pharmacists are particularly considered as one of the most accessible and reliable healthcare professionals.^5^,^6^ Many patients who are aiming to lose weight visit the community pharmacy to get weight management products or seek health advice. Furthermore, point-of-care devices that are commonly used for weight management are becoming readily available in community pharmacies.^7^,^8^ Therefore, community pharmacists have the opportunity to identify obese and overweight individuals who may or may not be considering weight management.^9^ Pharmacists can actively intervene and play a key role in obesity management. At the lower level of engagement, they can help patients in selecting an appropriate weight loss medication and counsel them about appropriate use of weight loss medications. At the higher level of engagement, pharmacists alone or in collaboration with physicians and dieticians can deliver advanced weight management services (WMSs). In the provision of these services, the pharmacist could use point-of-care devices, review the patient health history, assess the patient’s readiness to change an undesirable health behavior, offer motivational behavioral counseling, design a pharmacotherapeutic plan, and monitor the safety and efficacy of weight loss medications.^8–16^

Qatar is an independent state in the Middle East, situated in a peninsula that extends into the Arabian Gulf.^17^ According to a report published in the Lancet in 2014, Qatar is considered among the top five countries in the world with the prevalence of overweight and obese adults.^2^ Qatar has also the highest prevalence of obese men (44%) in the MENA region and it ranks third in the region in terms of prevalence of obesity in women (55%).^2^ Furthermore, obesity prevalence in school students aged 5–19 years was 40.4% in Qatar for the period of 2015–2016.^18^ In response to the obesity epidemic and its associated risks, the government of Qatar has introduced several initiatives and strategies such as the National Sport Day, the Qatar National Nutrition and Physical Activity Action Plan (2011–2016), and National Obesity Center.^19–22^ Currently there are over 1000 pharmacists practicing in community pharmacies in Qatar.^23^ The potential to utilize Qatar pharmacists in achieving the goals of these weight management initiatives has not been highlighted.

The objectives of this study were to explore Qatar community pharmacists’:

- practice experiences about the weight management services (WMSs) they currently offer in community pharmacies, if any.

- attitudes about pharmacist’s role in the provision of WMSs.
- perceived competence and training needs for the provision of WMSs.
- perceived barriers to the implementation of WMSs in community pharmacies.

## Methods

### Study Design

We used a two-phase mixed-method explanatory sequential design: (1) collection and analysis of quantitative data (cross-sectional survey); (2) collection and analysis of qualitative data (individual and focus group interviews). Qualitative data were collected using a phenomenological approach through individual face-to-face interviews and focus group discussions. The phenomenological approach was selected to explore the essence of community pharmacists’ common experiences in providing WMSs. Specifically, descriptive phenomenology was applied to investigate, analyze, and explain the role of community pharmacists in WMSs while preserving the richness, breadth, and depth of this phenomenon in order to obtain a “near-real view” of it.^24–26^ Finally, the quantitative and qualitative data sets were integrated for triangulation.

### Phase 1: Quantitative Phase

#### Participants

Eligible participants for this study were community pharmacists who provided patient care in their respective outlets.

#### Survey Instrument

A self-administered questionnaire was used to solicit responses from the participants. A thorough literature review was conducted to identify any published and validated questionnaires that can answer the study objectives. Retrieved questionnaires were evaluated and questions were adapted with modification to pharmacy practice context in Qatar.^27–30^ Questions that are aligned with the study objectives were adapted and modifications were done in relation to culture, clarity of language, and nature of the practice. The draft questionnaire was reviewed for face and content validity by an expert panel of experienced faculty at College of Pharmacy, Qatar University, as well as among a sample of 10 community pharmacists for clarity of questions, relevance of items, and time for completion. For each of the three sections: current involvement in WMSs, attitudes towards the provision of WMSs and perceived competence, Cronbach’s alpha coefficient was computed in order to determine the internal consistency of the items within each construct. The Cronbach’s alpha coefficient exceeded 0.90 for all the three sections (0.945, 0.924, 0.947, respectively) and overall (0.953).

#### Survey Implementation

The Qatar’s Ministry of Public Health (MOPH) database of community pharmacists was used as a sampling frame to randomly select the study participants. All selected pharmacists (n=600) were contacted and invited to participate in the study. An email containing the survey URL was sent to the selected pharmacists through SurveyMonkey^®^ platform. Reminders were sent via email at 2 to 4-weekly intervals.

#### Sample Size Calculation

At the time of the study, approximately 1200 community pharmacists were practicing in Qatar. With a margin of error of 5% and a confidence level of 95%, the minimum sample size required was calculated as 292 pharmacists. The target sample size was 600 pharmacists to account for any non-response.

#### Data Analysis

All data were analyzed using the Statistical Package for SPSS^®^ version 24 (IBM Corp, Armonk, NY). Descriptive statistics were computed for all the study variables using mean± standard deviation [SD] for normally distributed continuous variables and frequencies and percentages for categorical variables. For each participant, the average score for current involvement, attitudes and perceived competence was computed by taking the average of the respondent’s answers on all questions answered under each section. For example, each question related to involvement had a 5-point Likert scale answers (1=never, 2=rarely, 3=sometimes, 4=often, and 5=always). The average score for each participant was then computed as described above. The scores ranged between 1 and 5 with higher numbers indicating higher involvement, attitude, or perceived competence. The association between those scores and demographic variables was assessed using the independent *t*-test or one-way ANOVA depending on the number of groups/categories in the demographic variable and Pearson’s correlation for association with age. Moreover, nonparametric alternatives for the *t*-test, one-way ANOVA, and Pearson’s correlation were performed as a sensitivity analysis since the distribution of the scales of attitudes and perceived competence showed mild deviation from normality. A p-value of 0.05 or less was considered statistically significant.

### Phase 2: Qualitative Phase

#### Participants and Recruitment

All participants from Phase 1 who indicated interest to participate in the focus group interviews were pooled into a database that served as the sampling frame for the qualitative phase. Thus, a convenience sampling approach was used. Community pharmacists who consented to participate were sent invitation letters via email and were contacted through telephone a few days before the focus group session to remind them about the session and to confirm their attendance. To ensure participants freely discuss their views and experiences, the selected participants were grouped into homogenous focus groups based on their practice setting (chain vs independent pharmacy). In case of a low number of attendances for a particular focus group, direct face-to-face interviews were conducted with participants. The guidelines for conducting phenomenological research were applied including: 1) transcription, 2) bracketing and the phenomenological reduction, 3) listening to the interview for a sense of the whole, 4) delineating units of general meaning, 5) delineating units of meaning relevant to the research question, 6) training independent researchers to verify the units of relevant meaning, 7) eliminating redundancies, 8) clustering units of relevant meaning, 9) determining themes from clusters of meaning, 10) writing a summary for each interview, 11) returning to the participant with the summary and themes, 12) modifying themes and summary, 13) identifying general and unique themes for all the interviews, 14) contextualization of themes and, 15) composite summary.^31^

#### Focus Group Guide Development

For standardized and systematic data collection, focus group sessions were conducted using a semi-structured interview guide. The questions in the interview guide were developed primarily based on the survey results from Phase 1, on the study objectives, and published research on community pharmacy-based weight management.^32–34^To ensure validity, the interview guide was reviewed by faculty in pharmacy practice with expertise in qualitative or mixed-method research and by a purposive sample of pharmacists. This was followed by pilot interviews with four pharmacists who were excluded from the qualitative phase. Suggested changes were made to the final version of the guide.

#### Focus Group Sessions Structure

Focus group interviews were conducted with community pharmacists in Qatar at Qatar University. Each focus group session was about 60–120 minutes and comprised of 6–8 participants. Focus group sessions were conducted in English as English is one of the main languages for communication in Qatar. Each session involved one moderator (MH: female faculty member and project leader, PharmD degree holder with experience in pharmacy practice research) and one recorder (SM or NZ: BSc (Pharm) degree holder and female student researchers). The researchers did not have any established relationships with the participants and have been trained in qualitative and phenomenology research. The moderator facilitated the session and ensured equal opportunities for participants to discuss their viewpoint. The focus group sessions were audio-taped for transcribing purposes. The recorder took notes of the sessions including the quotes of the participants, the tone of the discussion, the order in which participants speak and any non-verbal expressions of the participants. At the end of each focus group, participants were given the opportunity to have further comments. After each focus group session, the research team had a debriefing session to validate the participants seating organization and coding, and to make recommendations for improving consequent focus group sessions.^35^ Data were collected until saturation.

#### Data Transcription and Analysis

The focus group sessions were audio-recorded and transcribed verbatim with use of the recorder’s notes from each session by one of the investigators and verified by another team member. The transcripts were shared with the participants for feedback, if any. Qualitative data analysis was carried out in parallel with data collection. This analytical process allowed the research team to continually check the collected data and to pursue emerging questions for subsequent focus groups.^36^ Transcripts were reviewed multiple times by the study team, and a code list was generated based on emerging patterns. Using thematic content analysis, two study investigators (SM, NZ) broke the text into small units of meaning or ideas or words, and independently coded the transcripts into themes and subthemes.^36^,^37^ Themes and subthemes were compared for similarities and differences between team members. Consensus was achieved through face-to-face discussions. The study results were shared with the study participants through email in case they wanted to offer any feedback. Finally, integration and triangulation of quantitative and qualitative data sets was undertaken. This phase of the study is conducted and reported in accordance with the Consolidated Criteria for Reporting Qualitative Studies (COREQ).^38^

### Ethical Considerations

The study was approved by Qatar University Institutional Review Board (approval number: QU-IRB 917-E/18). Written Informed consent was obtained from pharmacists who participated in the interviews. Pharmacists who filled the survey electronically signed consent form. Consent included publication of anonymized responses.

## Results

### Phase 1: Quantitative Data

The survey was kept open from April 2018 to June 2019 due to very slow response despite multiple periodic reminders (Supplementary File 1). During the data collection period, 354 surveys were collected. Eighty-four survey attempts contained no responses to the survey questions and were therefore excluded. Therefore, 270 respondents completed the survey and were included in the final study analysis (response rate, 45%). Table 1 presents the sociodemographic and practice characteristics of the community pharmacists.

**Table 1:** Socio-Demographic and Community Pharmacy Characteristics

Characteristic	Frequency (Percent)
**Age (N=247)** Mean (SD)	34.38 (6.3)
**Male Gender (N=270)**	153 (56.7%)
**Country of origin (N=270)**	
Qatar	2 (0.7%)
Egypt	119 (44.1%)
India	94 (34.8%)
Jordan	12 (4.4%)
Palestine	9 (3.3%)
Philippines	17 (6.3%)
Sudan	4 (1.5%)
Syria	5 (1.9%)
Pakistan	5 (1.9%)
Other^a^	3 (1.1%)
**Highest pharmacy degree (N=267)**	
B.Pharm/BSc Pharm	223 (83.5%)
PharmD	16 (6.0%)
MPharm	22 (8.2%)
MSc/MPhil	4 (1.5%)
Ph.D.	2 (0.8%)
**Country awarding highest pharmacy degree (N=268)**	
Egypt	121 (45.1%)
India	96 (35.8%)
Jordan	16 (6.0%)
Palestine	1 (0.4%)
Philippines	15 (5.6%)
Sudan	2 (0.8%)
Syria	4 (1.5%)
Pakistan	6 (2.2%)
Other^b^	7 (2.6%)
**Years of practice in Qatar (N=268)**	
Less than 5 years	141 (52.6%)
5–10 years	94 (35.1%)
11–15 years	17 (6.3%)
16–20 years	13 (4.9%)
More than 20 years	3 (1.1%)
**Position in the pharmacy (N=270)**	
Pharmacist in training	6 (2.2%)
Staff pharmacist	172 (63.7%)
Pharmacy supervisor	26 (9.6%)
Pharmacy manager	65 (24.1%)
Pharmacy owner	1 (0.4%)
**Community pharmacy type (N=270)**	
Independent single pharmacy	107 (39.6%)
Chain pharmacy	163 (60.4%)
**Community pharmacy location (N=269)**	
Pharmacy located in a shopping mall or supermarket	48 (17.8%)
Pharmacy located in a private clinic	53 (19.7%)
Pharmacy located in a private hospital	10 (3.7%)
Pharmacy located in a gas station	29 (10.8%)
Community pharmacy located in other places	129 (48.0%)
**Average number of pharmacists working during one shift (N=269)**	
1	200 (74.3%)
More than 1	69 (25.7%)
**Average number pharmacy technicians working during one shift (N=269)**	
0	90 (33.5%)
1	143 (53.2%)
More than 1	36 (13.4%)

**Notes**: ^a^Australia, Greece, Nepal. ^b^United States of America, Australia, Greece, United Kingdom, United Arab Emirates, Malaysia.

Over 80% of the respondents reported that medications, herbs, and dietary supplements for weight loss are available in their pharmacy. The most commonly dispensed weight loss medications by the pharmacists were orlistat (91.0%) and liraglutide (51.1%). Around half of the respondents indicated that one to three times weekly is the average time per week that weight loss medications, herbs or dietary supplements are dispensed (56.0%) and that weight management consultations are offered (58.6%).

The community pharmacists’ type and level of involvement in the provision of WMSs is described in Table 2. More than 60% of the respondents indicated that they rarely or never perform nine out of the 25 identified weight management services. For instance, 83.8% and 72.1% of the pharmacists rarely or never measured patients’ waist circumference or their BMI, respectively. On the other hand, more than one-half of the pharmacists indicated that they often or always explain the risks associated with overweight and obesity (56.2%), recommend weight loss medications, herbs, or dietary supplements (52.4%), and counsel about their proper use and/or side effects (56.9%). The average involvement score was 2.55±0.77. This score was significantly higher among pharmacists from chain pharmacies when compared to those from independent pharmacies (2.6±0.4 vs 2.4±0.79, p=0.040). No significant associations were found with other demographic variables.

**Table 2:** Current Community Pharmacist Involvement in Weight Management Services

How Frequently Do You Undertake the Following Weight Management Activities in Your Pharmacy?					
Statement	Frequency (Percent)				
	Never/1	Rarely/2	Sometimes/3	Often/4	Always/5
Measure patient’s weight (N=266)	107 (40.2%)	71 (26.7%)	51 (19.2%)	21 (7.9%)	16 (6.0%)
Measure patient’s height (N=265)	160 (60.4%)	58 (21.9%)	26 (9.8%)	12 (4.5%)	9 (3.4%)
Measure patient’s waist circumference (N=265)	179 (67.5%)	43 (16.3%)	28 (10.6%)	7 (2.6%)	8 (3.0%)
Calculate patient’s body mass index (BMI) (N=266)	127 (47.7%)	65 (24.4%)	47 (17.7%)	17 (6.4%)	10 (3.8%)
Estimate patient’s body fat percentage (N= 266)	185 (69.5%)	36 (13.5%)	30 (11.4%)	11 (4.1%)	4 (1.5%)
Measure patient’s blood cholesterol (N=266)	205 (77.1%)	29 (10.9%)	23 (8.6%)	5 (1.9%)	4 (1.5%)
Measure patient’s blood glucose (N=265)	160 (60.4%)	49 (18.5%)	37 (14.0%)	12 (4.5%)	7 (2.6%)
Measure patient’s blood pressure (N=263)	150 (57.0%)	48 (18.3%)	42 (16.0%)	13 (4.9%)	10 (3.8%)
Identify patient or medication related factors that may contribute to weight gain (N=266)	28 (10.5%)	49 (18.4%)	94 (35.4%)	58 (21.8%)	37 (13.9%)
Explain the risks associated with overweight and obesity (N=267)	14 (5.2%)	35 (13.1%)	68 (25.5%)	85 (31.8%)	65 (24.4%)
Offer weight management related brochures or written educational information (N=267)	66 (24.7%)	71 (26.6%)	63 (23.6%)	42 (15.7%)	25 (9.4%)
Evaluate obese/overweight patient readiness to change behavior in relation to weight management (N=265)	57 (21.5%)	66 (24.9%)	84 (31.7%)	37 (14.0%)	21 (7.9%)
Establish a target goal for weight range for overweight/obese patients (N=263)	76 (28.9%)	76 (28.9%)	65 (24.7%)	31 (11.8%)	15 (5.7%)
Estimate the daily caloric requirements for overweight/obese patients (N=263)	122 (46.4%)	62 (23.6%)	48 (18.2%)	22 (8.4%)	9 (3.4%)
Advice on a healthy diet to achieve weight loss (N=226)	17 (6.4%)	42 (15.8%)	67 (25.2%)	69 (25.9%)	71 (26.7%)
Review patient physical activity and design a physical activity plan (N=264)	69 (26.2%)	50 (19.0%)	68 (25.9%)	41 (15.6%)	35 (13.3%)
Recommend weight loss medications, herbs or dietary supplements (N=263)	26 (9.9%)	24 (9.2%)	75 (28.5%)	69 (26.2%)	69 (26.2%)
Counsel about the proper use and/or side effects of weight loss medications, herbs or dietary supplements (N=267)	21 (7.9%)	29 (10.9%)	65 (24.3%)	65 (24.3%)	87 (32.6%)
Recommend weight loss supplies (eg, diet foods, fat burning devices, steps counting devices, etc …) (N=267)	24 (9.0%)	43 (16.1%)	71 (26.6%)	71 (26.6%)	58 (21.7%)
Counsel about the proper use of and/or side effects of weight loss supplies (eg, diet foods, fat burning devices, steps counting devices, etc …) (N=266)	20 (7.5%)	39 (14.7%)	66 (24.8%)	69 (25.9%)	72 (27.1%)
Counsel about weight monitoring for patients who are taking medications that can cause weight gain (N=264)	25 (9.5%)	50 (18.9%)	79 (29.9%)	56 (21.2%)	54 (20.5%)
Monitor patient adherence to weight loss medications, herbs or dietary supplements (N=267)	76 (28.5%)	56 (21.0%)	79 (29.5%)	41 (15.4%)	15 (5.6%)
Monitor patient adherence to nutritional/physical activity advice (N=263)	68 (25.9%)	67 (25.5%)	79 (30.0%)	34 (12.9%)	15 (5.7%)
Review and monitor patient’s progress in relation to weight management (N= 264)	71 (26.9%)	79 (29.9%)	64 (24.2%)	36 (13.6%)	14 (5.4%)
Refer overweight/obese patients to other healthcare professionals and specialist services where appropriate (eg, nutritionist, bariatric specialist, etc …) for weight management purposes (N=267)	34 (12.7%)	58 (21.7%)	82 (30.7%)	62 (23.3%)	31 (11.6%)

Table 3 shows the community pharmacists’ attitudes towards the provision of WMSs. The majority of the respondents agreed or strongly agreed to all the nine attitudinal statements. The average attitude score was 4.10±0.71. This average score was significantly lower among pharmacists originating from Egypt (mean score 3.93±0.75) when compared to pharmacists from India (mean score 4.23±0.64) and pharmacists from other countries of origin (mean score 4.60±0.33) (p=0.039). Additionally, the attitude score was significantly higher among pharmacists with MPharm degree (mean score 4.62±0.46) as compared to those with BPharm/BSc Pharm degree (mean score 4.05±0.71) and those with PhD (mean score 3.5±0.55) (p=0.005).

**Table 3:** Community Pharmacists’ Attitudes Towards the Provision of Weight Management Services

What is Your Extent of Agreement with the Following Statements?					
Statement	Frequency (percent)				
	Strongly Disagree/1	Disagree/2	Neutral/3	Agree/4	Strongly Agree/5
Pharmacists should provide information and recommendations on weight loss medications, herbs and dietary supplements (N=261)	5 (1.9%)	2 (0.8%)	15 (5.7%)	113 (43.3%)	126 (48.3%)
Pharmacists should collaborate with other healthcare providers (eg physicians, dietitians, exercise physiologist or behavioral psychologist, etc …) to help overweight/obese patients in losing weight (N=261)	7 (2.7%)	1 (0.4%)	27 (10.4%)	104 (39.8%)	122 (46.7%)
Pharmacists should offer nutritional/dietary advice to help overweight/obese patients in losing weight (N=262)	4 (1.5%)	13 (5.0%)	38 (14.5%)	106 (40.5%)	101 (38.5%)
Pharmacists should offer physical activity recommendations to help overweight/obese patients in losing weight (N=262)	3 (1.1%)	17 (6.5%)	39 (14.9%)	103 (39.3%)	100 (38.2%)
Pharmacists should offer motivational/behavioral counseling to help overweight or obese patients in losing weight (N=262)	6 (2.3%)	4 (1.5%)	33 (12.6%)	120 (45.8%)	99 (37.8%)
Pharmacists should be involved in setting the optimal weight goal in overweight/obese patients (N=262)	3 (1.1%)	16 (6.1%)	42 (16.0%)	117 (44.7%)	84 (32.1%)
Pharmacists should be involved in monitoring weight related outcomes in overweight/obese patients (N=262)	4 (1.5%)	14 (5.3%)	44 (16.8%)	121 (46.2%)	79 (30.2%)
Pharmacists should use point-of-care devices in the pharmacy (e.g: weighing scale, blood pressure monitors, cholesterol testing etc …) (N=261)	6 (2.3%)	16 (6.1%)	38 (14.6%)	114 (43.7%)	87 (33.3%)
Pharmacists should assess cardiovascular risk in at risk patients such as obese and overweight patients (N=261)	7 (2.7%)	12 (4.6%)	41 (15.7%)	123 (47.1%)	78 (29.9%)

Table 4 shows community pharmacists’ perceived barriers for providing WMSs. A large proportion of the participants agreed or strongly agreed that pharmacist’s perceived difficulty in being able to adequately follow-up with the patient (80.7%), lack of private consultation area in the pharmacy (75.7%), and lack of pharmacist time for counseling (75.2%) are barriers for implementing WMSs.

**Table 4:** Perceived Barriers for Implementing Weight Management Services

What is Your Extent of Agreement with the Below Factors Being Considered as Barriers for Implementing Weight Management Services in the Community Pharmacy?					
Statement	Frequency (Percent)				
	Strongly Disagree/1	Disagree/2	Neutral/3	Agree/4	Strongly Agree/5
Lack of pharmacist time for counselling (N=258)	10 (3.9%)	15 (5.8%)	39 (15.1%)	122 (47.3%)	72 (27.9%)
Lack of patient demand for weight management services (N=256)	9 (3.5%)	34 (13.3%)	54 (21.1%)	120 (46.9%)	39 (15.2%)
Lack of or inadequate reimbursement/financial compensation for pharmacists in relation to weight management (N=258)	4 (1.6%)	18 (7.0%)	49 (19.0%)	105 (40.6%)	82 (31.8%)
Lack of private consultation area in the pharmacy (N=259)	4 (1.5%)	14 (5.4%)	45 (17.4%)	111 (42.9%)	85 (32.8%)
Lack of patient’s awareness of the pharmacist’s expertise in relation to weight management (N=257)	4 (1.6%)	22 (8.6%)	44 (17.1%)	120 (46.6%)	67 (26.1%)
Patient’s beliefs that obesity is controllable without medications (N=258)	9 (3.5%)	40 (15.5%)	54 (20.9%)	119 (46.1%)	36 (14.0%)
Lack of or inadequate pharmacist’s knowledge in relation to weight management (N=259)	6 (2.3%)	59 (22.8%)	51 (19.7%)	106 (40.9%)	37 (14.3%)
Lack of pharmacist’s interest to provide weight management services in the pharmacy (N=259)	11 (4.2%)	68 (26.3%)	50 (19.3%)	88 (34.0%)	42 (16.2%)
Pharmacist perceived difficulty in being able to adequately follow-up with the patient (N=259)	5 (1.9%)	11 (4.2%)	34 (13.2%)	140 (54.1%)	69 (26.6%)
Lack of management support in relation to provision of weight management services in the pharmacy (N=258)	2 (0.8%)	23 (8.9%)	52 (20.1%)	116 (45.0%)	65 (25.2%)
Lack of willingness among patients to work at losing weight (N=258)	5 (1.9%)	19 (7.4%)	60 (23.3%)	125 (48.4%)	49 (19.0%)
Patient opinions about obesity not as a disease (N=254)	4 (1.6%)	20 (7.9%)	55 (21.6%)	125 (49.2%)	50 (19.7%)

In relation to the community pharmacists’ perceived competence for providing WMSs, more than 60% of the respondents indicated that they are fully competent in seven out of 24 listed competency areas (Table 5). However, around one-third of the pharmacists reported not being competent in estimating patient’s body fat percentage (30.3%), measuring patient’s blood cholesterol (32.2%), and estimating the daily caloric requirements for overweight/obese patients (31.0%). The average perceived competence score was 2.36±0.46. This average score was significantly higher among those with 5–10 years of experience (mean score 2.46±0.39) as compared to those with less than 5 years of experience (mean score 2.31± 0.49) and those with 16–20 years of experience (mean score 2.17±0.45) (p=0.036).

**Table 5:** Perceived Competence for Providing Weight Management Services

Please Rate Your Self-Perceived Competence in Relation to Provision of the Weight Management Services Below on a 3-Point Likert Scale			
Statement	Frequency (Percent)		
	Not Competent/1	Partiality Competent/2	Fully Competent/3
Measure patient’s weight (N=256)	24 (9.4%)	52 (20.3%)	180 (70.3%)
Measure patient’s height (N=254)	34 (13.4%)	56 (22.0%)	164 (64.6%)
Measure patient’s waist circumference (N=256)	47 (18.4%)	80 (31.2%)	129 (50.4%)
Calculate patient’s body mass index (BMI) (N=255)	24 (9.4%)	73 (28.6%)	158 (62.0%)
Estimate patient’s body fat percentage (N=254)	77 (30.3%)	97 (38.2%)	80 (31.5%)
Measure patient’s blood cholesterol (N=255)	82 (32.2%)	82 (32.2%)	91 (35.6%)
Measure patient’s blood glucose (N=254)	36 (14.2%)	58 (22.8%)	160 (63.0%)
Measure patient’s blood pressure (N=255)	31 (12.2%)	61 (23.9%)	163 (63.9%)
Identify patient or medication related factors that may contribute to weight gain (N=254)	12 (4.7%)	108 (42.5%)	134 (52.8%)
Explain the risks associated with overweight and obesity (N=255)	7 (2.7%)	73 (28.7%)	175 (68.6%)
Evaluate obese/overweight patient readiness to change behavior in relation to weight management (N=256)	25 (9.8%)	137 (53.5%)	94 (36.7%)
Establish a target goal for weight range for overweight/obese patients (N=254)	39 (15.4%)	143 (56.3%)	72 (28.3%)
Estimate the daily caloric requirements for overweight/obese patients (N=255)	79 (31.0%)	121 (47.4%)	55 (21.6%)
Advice on a healthy diet to achieve weight loss (N=255)	18 (7.1%)	100 (39.2%)	137 (53.7%)
Review patient physical activity and design a physical activity plan (N=253)	40 (15.8%)	126 (49.8%)	87 (34.4%)
Recommend weight loss medications, herbs or dietary supplements (N=253)	13 (5.2%)	95 (37.5%)	145 (57.3%)
Counsel about the proper use and/or side effects of weight loss medications, herbs or dietary supplements (N= 254)	8 (3.2%)	90 (35.4%)	156 (61.4%)
Recommend weight loss supplies (eg, diet foods, fat burning devices, steps counting devices, etc …) (N=254)	17 (6.7%)	98 (38.6%)	139 (54.7%)
Counsel about the proper use of and/or side effects of weight loss supplies (eg, diet foods, fat burning devices, steps counting devices, etc …) (N=254)	15 (5.9%)	105 (41.3%)	134 (52.8%)
Counsel about weight monitoring for patients who are taking medications that can cause weight gain (N=254)	23 (9.0%)	112 (44.1%)	119 (46.9%)
Monitor patient adherence to weight loss medications, herbs or dietary supplements (N=254)	35 (13.8%)	114 (44.9%)	105 (41.3%)
Monitor patient adherence to nutritional/physical activity advice (N=253)	39 (15.4%)	121 (47.8%)	93 (36.8%)
Review and monitor patient’s progress in relation to weight management (N=254)	36 (14.2%)	122 (48.0%)	96 (37.8%)
Refer overweight/obese patients to other healthcare professionals and specialist services where appropriate (eg, nutritionist, bariatric specialist, etc …) for weight management purposes (N=253)	25 (9.9%)	100 (39.5%)	128 (50.6%)

Furthermore, approximately 90% of the respondents indicated that they did not complete any weight-management-related training and/or continuing professional development (CPD). However, the majority (89.5%) are interested in receiving weight-management-related training and/or CPD in the future.

### Phase 2: Qualitative Data

**Figure 1:**
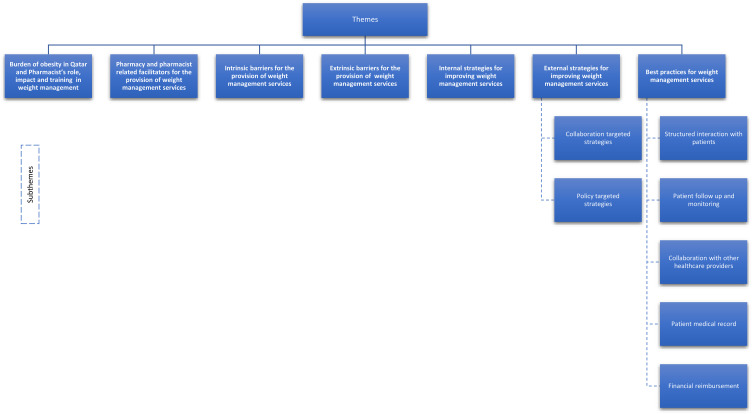
Conceptual diagram of key themes and subthemes.

Three focus groups and two face-to-face individual interviews were conducted between June 2019 to October 2019 (Supplementary Table 1). Of 270 pharmacists who completed the quantitative survey, 50 pharmacists consented to participate in the focus groups, and 15 pharmacists eventually attended. Interviews lasted between 45 and 60 minutes. Data saturation based on both face-to-face individual interviews and focus groups was achieved as no new information was elicited from participants. No repeat interviews were conducted. Seven themes emerged in relation to pharmacists’ experiences and perceptions about WMSs, barriers and facilitators (Figure 1). Quotes are included in the text to contextualize the results. Themes and subthemes were shared with study participants.

#### Theme 1: Burden of Obesity in Qatar and Pharmacist’s Role, Impact, and Training in Weight Management

The participants perceived that obesity is a crucial public health issue in Qatar. They attributed the causes of obesity to the sedentary lifestyle and unhealthy diet of Qatar’s community. They also mentioned that the lack of awareness about the complications of obesity among Qatar’s high school students could be a contributing factor for obesity in the country. The participants had a consensus on the high number of performed bariatric surgeries in Qatar despite the concerns about the potential risks of these surgeries.

“In Doha, obesity is a public health concern because we are seeing patients coming asking for weight reduction products” P14

The participants indicated that pharmacists play an important role in providing WMSs in the community. They summarized the activities undertaken to perform the role as follows: building rapport with the patient, gathering patient’s information, recommending a weight loss product, and counseling on healthy lifestyle and appropriate use of the weight loss product. They also recognized pharmacist’s role in promoting health and preventing diseases. Some participants indicated that they frequently measure patients’ blood glucose, blood pressure, and BMI in their community pharmacies. They also considered that patient monitoring, assessing outcomes, offering follow up, and referring to other healthcare providers when necessary, constitute part of their duties as community pharmacists.

“I need to clarify to him what is the reason for his obesity, that is why I need to check all of his history” P2

“As I told you, I give lifestyle recommendations. Then, I offer medications” P1

“You have to advise the patient about how to use the medication, when to use it, and about its side effects, like orlistat that causes diarrhea” P5

Furthermore, the interviewees agreed that the provision of WMSs has an important impact on pharmacists, pharmacy practice, and patients. Overall, they perceived that the provision of WMSs can enhance their self-satisfaction and self-confidence through enriching their knowledge and experience. On the pharmacy level, pharmacist-offered WMSs can improve the customers’ loyalty to the pharmacy as well as increase the pharmacy sales and commercial benefits. Most importantly, the participants believed that offering WMSs can contribute to the achievement of optimal weight loss goal in overweight and obese patients.

It feels good and excellent when you guide someone to something that he wants. You feel like you are a personal guide by giving him good instructions and he follows them, then he loses weight P9

“Offering weight management services can increase our level of knowledge and skills as pharmacists because we will search for the information and get updated” P12

“Sometimes you may save a patient’s life. Some patients may try a weight loss medication from the internet without knowing any information about it” P6

Yet, the participants acknowledged the diversity of Qatar community pharmacists who emerge from diverse countries and cultures as well as educational backgrounds, which can lead to discrepancies in their knowledge and skills level related to WMSs.

“Pharmacists are coming from different backgrounds and each one might have different perspectives. So, there are no common grounds” P2

However, the participants had divergence of opinion regarding their knowledge and skills in relation to the provision of WMSs. Some interviewees believed that they possessed the required competence to deliver WMSs, while others admitted lack of adequate competence in this area.

“Not everyone works in the same way. Some pharmacists have a high level of knowledge while others do not” P4

The participants generally perceived themselves as not fully competent and confident to provide a comprehensive and holistic weight management program. As one interviewee asserted: “I cannot run a whole program on weight management by myself as a pharmacist” P2

Furthermore, the majority of the participants interviewed indicated that they did not receive any weight management-related training and generally expressed interest in receiving a formal training on weight management. Training topics deemed essential included, but not restricted to, herbal products and supplements, healthy diet, exercise, counselling skills, weight management-related calculations, and evidence-based weight management medications.

I need training on diet like which diet is suitable for specific persons. I do not have much knowledge about the diet. I cannot give a patient a dietary plan to follow P9

“We need training on effective weight management medications” P5

#### Theme 2: Pharmacy and Pharmacist Related Facilitators for the Provision of WMSs

The interviewees identified several facilitators for the provision of WMSs in Qatar. They mentioned the availability of a wide range of weight loss products in Qatar community pharmacies, and the offering of free pharmacy services as pharmacy-related facilitators.

“There are too many options of weight loss products including herbs, medications, and machines” P11

“It is very easy to communicate with the pharmacist; patients do not pay money to get the information from us” P3

The location of the pharmacy was also identified as a facilitator to WMSs provision; pharmacists working in community pharmacies located in medical centers are more likely to collaborate with other healthcare providers.

“For me, I think it is easy because I am in a polyclinic where it is easy to discuss things with the doctors” P10

All of the participants believed that practicing for long time in the same pharmacy facilitates patients’ follow-up and builds their loyalty with the pharmacy. They also stated that online platforms, including social media and mobile applications, can play an important role in improving pharmacy–patient interaction and can make pharmacist counselling on weight management products more effective.

“For our pharmacy, we have a webpage, Facebook^®^ page, and WhatsApp^®^ number. Every day we have questions from patients, and we answer them continuously” P6

#### Theme 3: Intrinsic Barriers for the Provision of WMSs

The interviewees discussed several barriers for the provision of WMSs in community pharmacies in Qatar. Intrinsic barriers included lack of access to patients’ medical records and documentation in the pharmacy, lack of drug information resources, and lack of private area for counseling.

In addition, the interviewees highlighted multiple practical constraints that potentially restrain their ability to offer WMSs to overweight and obese patients. These constraints included lack of pharmacist time, lack of an adequate number of pharmacists working at any particular shift, high pharmacy-related workload, language barrier, and difficulty to follow-up and monitor patients. Other perceived barriers were lack of updated knowledge in relation to weight management, lack of motivation and interest for offering these services, and lack of collaboration with other healthcare providers.

“Here in Qatar, we have a language barrier, some people do not speak Arabic or English and you cannot speak freely with them” P14

“That is the problem because we do not have strong contact with other healthcare providers and there is no collaboration with them” P8

#### Theme 4: Extrinsic Barriers for the Provision of WMSs

The interviewed community pharmacists identified several extrinsic barriers for the provision of WMSs. They stated several important policy-related barriers for the provision of pharmacy-provided WMSs, including lack of support from pharmacy managers, lack of a formal professional pharmacy association in Qatar, and lack of financial reimbursement for pharmacy services.

“Pharmacy managers do not encourage pharmacists to be involved in weight management services” P2

The participants further discussed several challenges and barriers related to patients. These included the patients’ lack of awareness about pharmacist’s role in weight management, and lack of interest in WMSs offered by the pharmacist.

“Some patients are not interested to listen to an advice from the pharmacists about weight management because they do not appreciate our role” P10

The community pharmacists also believed that social media has been widely adopted by the public and negatively invades every aspect of their life. Patients consider online platforms as reliable sources for drug information and this can be a barrier for provision of WMSs. Furthermore, they are extremely influenced by the advertisements of weight loss products on these platforms as noted by one participant:

Actually, now the patients are driven by social media. So, you find today many patients are asking me the same question about a particular weight loss product because one influencer on Instagram® said something about it P15

#### Theme 5: Internal Strategies for Improving WMSs

Pharmacists proposed several internal strategies to improve WMSs. They include pharmacy and pharmacist-related strategies.

Establishing a documentation system for patients’ medical records, having a private area for counseling, setting up awareness campaigns in relation to obesity, using pictograms and translation programs for patients with language barriers, and increasing the number of pharmacists and technicians who work in each shift are among the several pharmacy-related strategies for improving WMSs in Qatar community pharmacies as proposed by the participants.

“Also, we need to have a documentation system for all pharmacies to know patient’s history and allow monitoring” P5

“I feel we must have a proper space in the pharmacy to talk with patients” P9

Most community pharmacists interviewed agreed on the suggestion that structured and regular training would promote their professional expertise in WMSs. Furthermore, they emphasized on involving community pharmacists in research, as well as utilizing social media to raise awareness about the complications of obesity and about the role of pharmacist in combating the epidemic of obesity. Participants noted that:

“We need the pharmacists to participate in research studies that are concerned about the community pharmacy practice” P3

#### Theme 6: External Strategies for Improving WMSs

Pharmacists suggested some external strategies to improve WMSs. They include collaborations and policy-related strategies.

#### Subtheme 6a: Collaboration-Targeted Strategies

Most pharmacists expressed the need for collaboration with various educational institutions, including Qatar University to help in establishing comprehensive weight management programs in Qatar.

“Qatar University can cooperate with us through running a weight management program provided by pharm D graduates” P15

Several pharmacists interviewed indicated that due to the complex nature of obesity, implementing a successful WMSs requires a multidisciplinary healthcare team approach.

Collaboration between the physician, nutritionist and pharmacist is helpful. The physician will diagnose, the nutritionist will create a dietary plan and the pharmacist will guide the patient on the appropriate weight loss products P9

#### Subtheme 6b: Policy-Targeted Strategies

In response to a question about policy-related strategies, the participants proposed establishing updated and unified guidelines for pharmacists to adapt for WMSs One participant pointed out that:

We must have a proper guideline by the Ministry of Health for all the community pharmacists as healthcare practitioners. So, we will have well revised and updated guidelines and we will be confident enough to provide information to the patients P9

#### Theme 7: Best Practices for WMSs

The community pharmacists involved in the FGDs and interviews recommended several best practices for WMSs in community pharmacies in Qatar. They are summarized below under different subthemes.

#### Subtheme 7a: Structured Interaction with Patients

The community pharmacists proposed that an ideal weight management program model should incorporate a structured interaction with patients that has various elements including building rapport, gathering information, individualizing weight management recommendations, counselling and education in relation to healthy lifestyle and appropriate use of weight loss products, offering educational brochures, and providing behavioral therapy.

“First, I need to open the conversation by breaking the ice, I don’t want to just provide information. I need to speak with him in a friendly way” P2

#### Subtheme 7b: Patient Follow Up and Monitoring

The pharmacists suggested that patient monitoring and follow up are essential components of any weight management practice model. They explained that this could be assisted by directly contacting patients and sending them text messages on a regular basis, in order to ensure the attainment and maintenance of optimal weight loss goals.

“As a part of the program, pharmacists can follow up with patients through calling them or sending them text messages” P6

They also recommended using social media platforms such as WhatsApp^®^, Facebook^®^, and Twitter^®^ to facilitate patient interaction, and they deemed this approach tremendously useful in any ideal weight management model.

We can have a unified online platform in the pharmacy by creating an online account or application with a username and password for each patient where they can get in contact with the pharmacists P5

#### Subtheme 7c: Collaboration with Other Healthcare Providers

Collaboration with other healthcare professionals, including physicians and dietitians was also highlighted as an essential part of any weight management practice model.

“I will refer him to a physician to do a blood test then to a dietician and trainer to have a plan for diet and exercise, then I will follow up with the team” P9

#### Subtheme 7d: Patient Medical Record Connecting Community Pharmacies to All Healthcare Institutions in Qatar

The participants recommended having an electronic medical record connecting community pharmacies to all healthcare institutions in Qatar.

“We can have an electronic system that is linked to other healthcare institutions in Qatar for documentation where it includes all patient’s related information” P12

#### Subtheme 7e: Financial Reimbursement for Providing WMSs

Finally, financial reimbursement was considered a key factor for pharmacists to implement effective WMSs. Payment for pharmacist was suggested to cover the extra workload that is associated with developing and maintaining weight management programs.

“The pharmacist should be paid for developing and running weight management programs” P4

## Discussion

This study is the first to explore the role of community pharmacists in the provision of WMSs in Qatar. The majority of the community pharmacies surveyed stocked weight loss medications, herbs or dietary supplements and dispensed them one to three times weekly. These findings are consistent with the high prevalence of obesity in Qatar with over 70% of the population being overweight or obese.^2^ These findings are also comparable to those of previous studies that targeted the role of community pharmacists in the provision of WMSs in Australia and UK.^27–29^

The findings of the current study reaffirm that Qatar community pharmacists are offering basic weight management services. Commonly reported practices include recommending weight loss products, counseling on appropriate use of the products, advising on healthy lifestyles to achieve weight loss, and explaining the risks associated with overweight and obesity. These findings are in line with those of previous studies of WMSs among community pharmacies in Scotland, Australia, and Kuwait.^27^,^28^,^30^

On the other hand, Qatar community pharmacists indicated that they are not able to provide comprehensive, holistic or advanced weight management programs. These results are in contrast to what has been published in Australia and UK where measurement of weight, height, BMI, and body fat is part of the pharmacist’s job responsibilities.^27^,^28^,^32^

Half of the study participants reported never or rarely offered weight-management-related brochures or written educational information. This result is consistent with previous studies among community pharmacists in Qatar where 78% of them never provided patients with breast cancer educational materials,^39^ and 49% never or rarely assisted tobacco smokers in quitting by offering them educational materials.^40^

Despite their basic WMSs, Qatar pharmacists recognize the burden that obesity is imposing on Qatar’s society, healthcare system, and economy. They attributed the causes of obesity in the country to Qatar’s sedentary lifestyle and unhealthy dietary habits which is consistent with published literature on the leading causes of obesity in the Gulf region.^41^,^42^ They also perceived that patients do not consider obesity as a metabolic disease and are still unaware of its complications. Other studies in the region have reported the lack of public awareness of the metabolic nature of obesity.^43^,^44^ Participants also considered that social media play a role in influencing patients’ perceptions and choices of weight loss modalities. In fact, the use of social media for marketing of medications is becoming a popular and alarming phenomenon nowadays where non-expert influencers or so-called bloggers have started sharing their medication use experience, promoting the use of illegal medications, and posting misleading information about medications.^45^,^46^ This highlights the important function that Qatar community pharmacists shall play in educating the public about the potential harms of using some social media platforms as reliable sources of medication information.

Qatar pharmacists have very positive attitudes towards their role in the provision of WMSs and they considered that these services have a great impact on patients, pharmacists, and pharmacy practice. These findings are very promising as community pharmacists are well positioned in the society to offer weight management services.^7^,^10–16^ Despite this, some pharmacists have raised a concern on the lack of public awareness about the role of community pharmacists in weight management in Qatar. Krska et al and Gray et al had reported similar results pertaining to patients’ lack of awareness of pharmacist’s role in weight management in the UK, and New Zealand.^29^,^32^,^33^

The interviewed pharmacists indicated their training needs such as evidence-based pharmacotherapy of weight management, post-partum weight management, and counseling skills. These results are comparable to what Um et al have reported in their Australian studies of what obesity experts, representatives of Australian professional pharmacy organizations, and Australian pharmacists perceived as the training needs of pharmacists.^34^,^47^ Collaborative efforts by the Qatar Ministry of Public Health and the Qatar University CPD program should be pursued to establish a formalized training program for weight management that is accredited by Qatar Council for Healthcare Practitioners (QCHP) for community pharmacists to empower them with the bundles of skills and knowledge that are required for the provision of effective WMSs.

Qatar community pharmacists perceived several barriers for the provision of WMSs. Top perceived barriers were lack of pharmacist time, lack of a private area for consultation, lack of financial compensation for pharmacists, lack of patient’s awareness of the pharmacist’s expertise in relation to weight management, inability of pharmacists to follow-up the patient, lack of managerial support, and language barriers. Some of these barriers are similar to the ones reported in previous studies that explored the role of pharmacist in weight management.^27^,^30^,^33^,^48^

For a better engagement of pharmacists in WMSs, Qatar pharmacists suggested several strategies to overcome the perceived barriers to provision of the service. Interestingly, Qatar pharmacists proposed the use of social media and mobile applications to increase the public awareness of pharmacist role in obesity management and to improve the pharmacist–patient interaction. In recent years, social media has become an increasingly used platform for communication with the public and for improving their knowledge and understanding of health issues of public health significance including cancer and obesity.^49^,^50^ In addition, mobile applications are also been increasingly utilized as tools for delivery of pharmacy services and may improve patient knowledge and quality of life.^51^ The use of translation programs and pictograms in patients with language difficulties was also suggested. Qatar is a multinational country that hosts over 80 nationalities with the majority coming from India, Nepal, and Bangladesh.^52^ Communication barrier between healthcare professionals and patients can lead to poor health outcomes. Use of pictograms supported by verbal instructions can be better understood by patients with low literacy than labels with written instructions or labels with verbal instructions in a language that the patient does not understand.^53^

Qatar pharmacists emphasized the importance of collaboration with other healthcare providers as an important element of any weight management program. There are several studies published in the literature that demonstrated the effectiveness, success, and feasibility of collaborative clinics.^8^ However, incorporating these interprofessional clinics in Qatar would necessitate thorough and detailed reviews of the healthcare infrastructure and system of the country, and would require engagement and commitment from all involved stakeholders.

To offer comprehensive and effective weight management services, the community pharmacists proposed an ideal weight management best practice model that encompasses structured interaction with patients, follow up and monitoring, collaboration with other healthcare providers, documentation system, and financial reimbursement. These practice components were demonstrated to be effective in previously published studies.^28^,^34^,^47^ However, there is need to test the effectiveness and feasibility of implementing this model in Qatar.

There are some study limitations that should be acknowledged in order to accurately interpret the findings. First, there is a possibility that social desirability bias could have overestimated the pharmacists’ self-reported provision of WMSs. Second, nonresponse bias could have occurred as some respondents included in the sample did not respond due to refusals to participate or the inability for being reached. However, based on the sociodemographic characteristics of respondents, we believe that the responses of study participants reflect the responses of Qatar community pharmacists, which confirm the external validity of the study results.

Third, the survey was administered in English only; therefore, some Arabic-speaking pharmacists might have faced some difficulties in understanding the survey questions in English. However, based on previous studies in Qatar, administering an English-based survey to Qatar pharmacists was not reported to be a problem.

## Conclusion

In conclusion, Qatar pharmacists have indicated very positive attitudes towards the provision of WMSs. Yet they are not able to provide comprehensive weight management programs and their WMSs are limited to counseling patients on weight loss medications, advising on healthy lifestyles to achieve weight loss and explaining the risks associated with obesity. This gap between Qatar community pharmacists’ attitudes and actual practice could be attributed to lack of pharmacist weight-management-related training and several anticipated barriers. Furthermore, Qatar pharmacists suggested developing an ideal weight management best practice model that encompasses the components of a comprehensive medication therapy management service. Before potential implementation, this model should be piloted and tested in Qatar for effectiveness in terms of weight loss for cost implications and for feasibility.

## Abbreviations

COREQ, Consolidated Criteria for Reporting Qualitative Studies; CPD, continuing professional development; CVDs, cardiovascular diseases; MENA, Middle East and North Africa; MOPH, Ministry of Public Health; UK, United Kingdom; US, United States; WHO, World Health Organization; WMSs, weight management services.
